# Supplementary material for: Inactivation of a CRF-dependent amygdalofugal pathway reverses addiction-like behaviors in alcohol-dependent rats
Source: Nat Commun. 2019 Mar 18;10:1238. doi: 10.1038/s41467-019-09183-0 (PMC6423296; doi:10.1038/s41467-019-09183-0)
Supplement: Supplementary file 1 — Supplementary Information [file 41467_2019_9183_MOESM1_ESM.pdf]

Inactivation of a CRF-dependent amygdalofugal pathway reverses addiction-like behaviors in alcohol-dependent rats

de Guglielmo et al.

Supplementary Information

Supplementary Notes

Inhibition of the CRF$^{CeA\text{-}SI}$ pathway does not affect alcohol drinking in dependent rats

Crh-Cre rats (*n* = 16) were bilaterally infused with AAV5-EF1a-DIO-NpHR-eYFP (*n* = 8) or AAV-DIO-eYFP (*n* = 8) in the CeA and unilaterally implanted with an optical fiber in the *substantia innominata* (SI; Supplementary Figure 5B). The escalation of alcohol intake was unaffected by the optogenetic inhibition of CRF$^{CeA\text{-}SI}$ terminals. Operant responding for alcohol remained high on both the laser ON and OFF days (Supplementary Figure 5C, black circles). The activation of the green laser in the SI in animals that were previously injected with the control virus, AAV-DIO-eYFP, in the CeA did not affect the escalation of alcohol intake during withdrawal (Fig. S5C, white circles). Finally, As shown in Supplementary Figure 5D, the inhibition of CRF$^{CeA\text{-}SI}$ terminals did not affect overall withdrawal severity or saccharin self-administration (Supplementary Figure 5E). At the end of the experiments, histological verification revealed incorrect optical fiber placements in two rats (Supplementary Figure 9C). Data from these animals were not included in the statistical analysis

Inhibition of the CRF$^{CeA\text{-}LH/pSTN}$ pathway does not affect alcohol drinking in dependent rats

Crh-Cre rats (*n* = 17) were bilaterally infused with AAV5-EF1a-DIO-NpHR-eYFP (*n* = 8) or AAV-DIO-eYFP (*n* = 9) in the CeA and unilaterally implanted with an optical fiber in the lateral hypothalamus/parasubthalamic nucleus (LH/pSTN; Supplementary Figure 6B). The escalation of alcohol intake was unaffected by the optogenetic inhibition of CRF$^{CeA\text{-}LH/pSTN}$ terminals. Operant responding for alcohol remained high on both the laser ON and OFF days (Supplementary Figure 6C, black circles). Activation of the green laser in the LH/pSTN in animals that were previously injected with the control virus, AAV-DIO-eYFP, in the CeA did not affect the escalation of alcohol intake during withdrawal (Supplementary Figure 6C, white circles). The inhibition of

$CRFCeA^{-LH/pSTN}$ terminals did not affect overall withdrawal severity or saccharin self-administration (Supplementary Figure 5D, E). At the end of the experiments, histological verification revealed incorrect optical fiber placements in three rats (Supplementary Figure 9D). Data from these animals were not included in the statistical analysis.

Inhibition of the $CRF^{CeA\text{-}PBN}$ pathway does not affect alcohol drinking in dependent rats

Crh-Cre rats (n=24) were bilaterally infused with AAV5-EF1a-DIO-NpHR-eYFP ($n = 14$) or AAV-DIO-eYFP ($n = 10$) in the CeA and unilaterally implanted with an optical fiber in the parabrachial nucleus (PBN, Supplementary Figure 7B). The escalation of alcohol intake was unaffected by the optogenetic inhibition of $CRF^{CeA\text{-}PBN}$ terminals. Operant responding for alcohol remained high on both the laser ON and OFF days (Supplementary Figure 7C, black circles). The activation of the green laser in the PBN in animals that were previously injected with the control virus, AAV-DIO-eYFP, in the CeA did not affect the escalation of alcohol intake during withdrawal (Supplementary Figure 7C, white circles). Finally, the inhibition of $CRF^{CeA\text{-}PBN}$ terminals did not affect overall withdrawal severity (Supplementary Figure 7D) or saccharin self-administration (Supplementary Figure 7E). At the end of the experiments, histological verification revealed incorrect optical fiber placements in four rats (Supplementary Figure 9E). Data from these animals were not included in the statistical analysis.

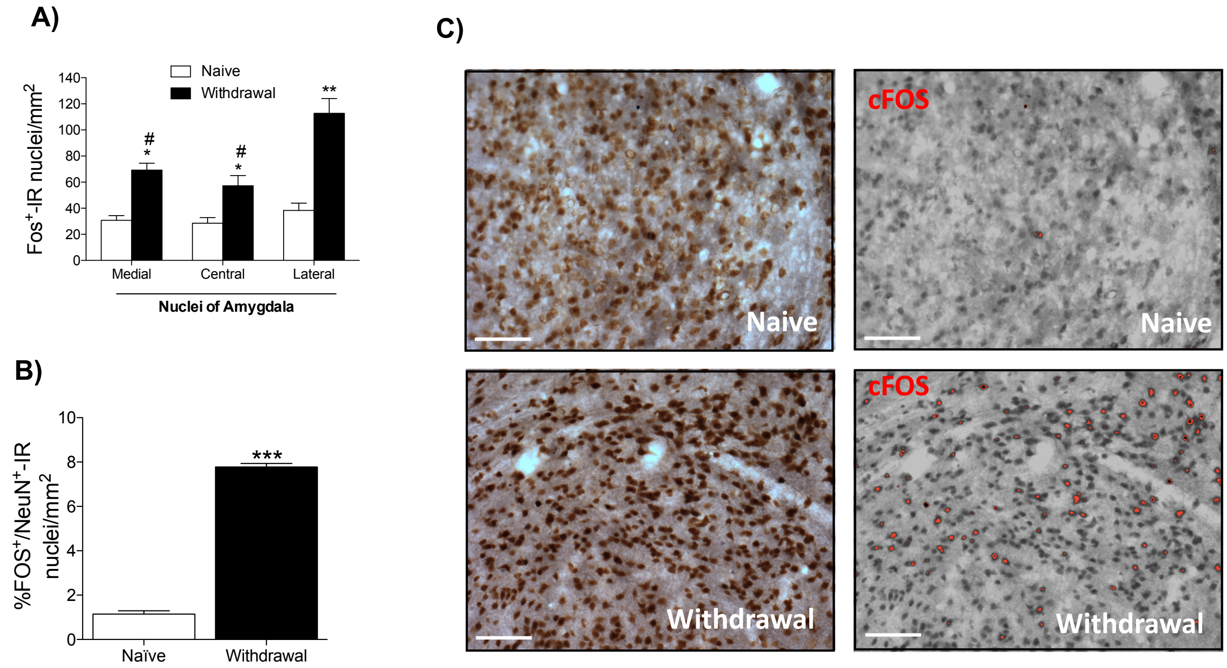

Supplementary Figure 1. (**A**) Fos expression during alcohol withdrawal in the three different nuclei of the amygdala. **p* < 0.05, ***p* < 0.05, *vs*. naive; $^{\#}p < 0.05$, *vs*. CeL (two-way ANOVA followed by Newman Keuls *post hoc* test). (**B**) Fos-NeuN co-localization in the CeA in naive and alcohol-withdrawn rats. ****p* < 0.001 (unpaired *t*-test). (**C**) Representative image of double immunohistochemistry Fos-NeuN in the CeA of rats in the naive and alcohol-withdrawal conditions. Data are expressed as ± SEM. Scale bar: 200 um.

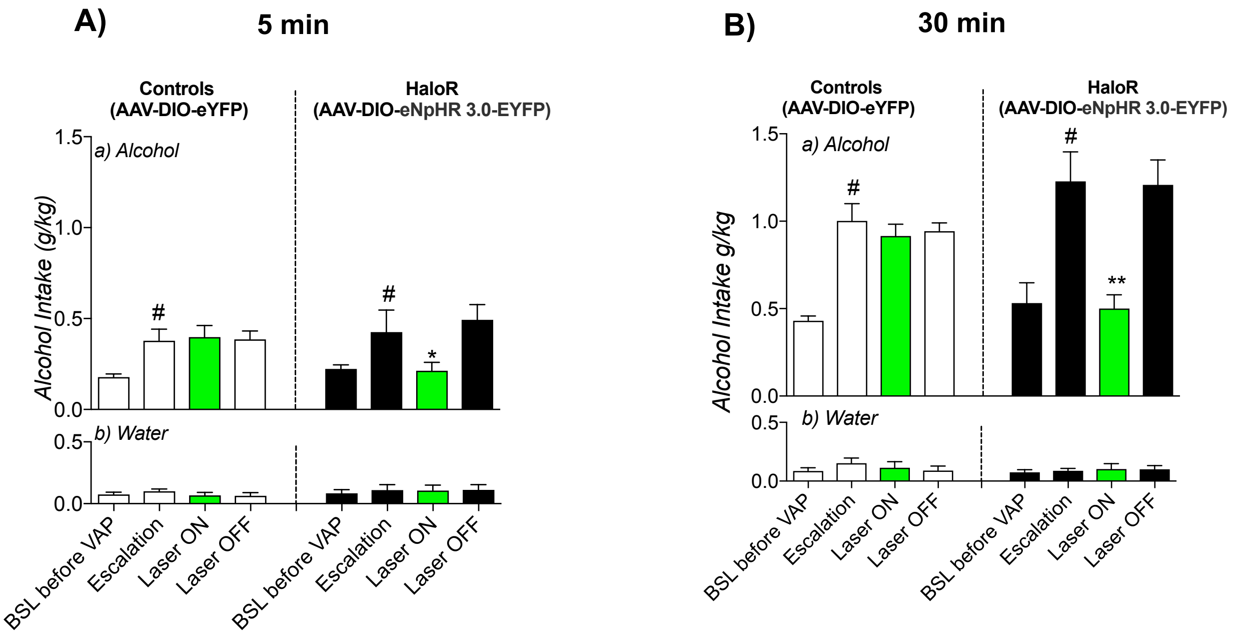

Supplementary Figure 2. Effect of optogenetic inhibition of CeA CRF neurons on alcohol (**a**) and water (**b**) self-administration in alcohol-dependent rats after 5 min (**A**) or 30 min (**B**) of optical inhibition. Activation of the green laser selectively reduced alcohol self-administration in NpHR-expressing rats. (**A**) There was a significant virus type × treatment interaction ($F_{2,28}$ = 3.39, $p < 0.05$), with the escalation of alcohol intake after chronic intermittent vapor exposure ($^{\#}p$ < 0.05, compared with intake before vapor exposure) and a selective reduction of alcohol intake in alcohol-dependent NpHR-expressing rats when the green laser was ON (*$p < 0.05$, compared with laser OFF). There were no effects of virus type or laser treatment on water self-administration, with no virus × treatment interaction. (**B**) For statistical results, see Fig. 3C. $^{\#}p <$ 0.05, *vs*. BSL pre-vapor; *$p < 0.05$, **$p < 0.01$, *vs*. escalation or laser OFF (mixed-factorial ANOVA followed by Newman Keuls *post hoc* test). Data are expressed as ± SEM.

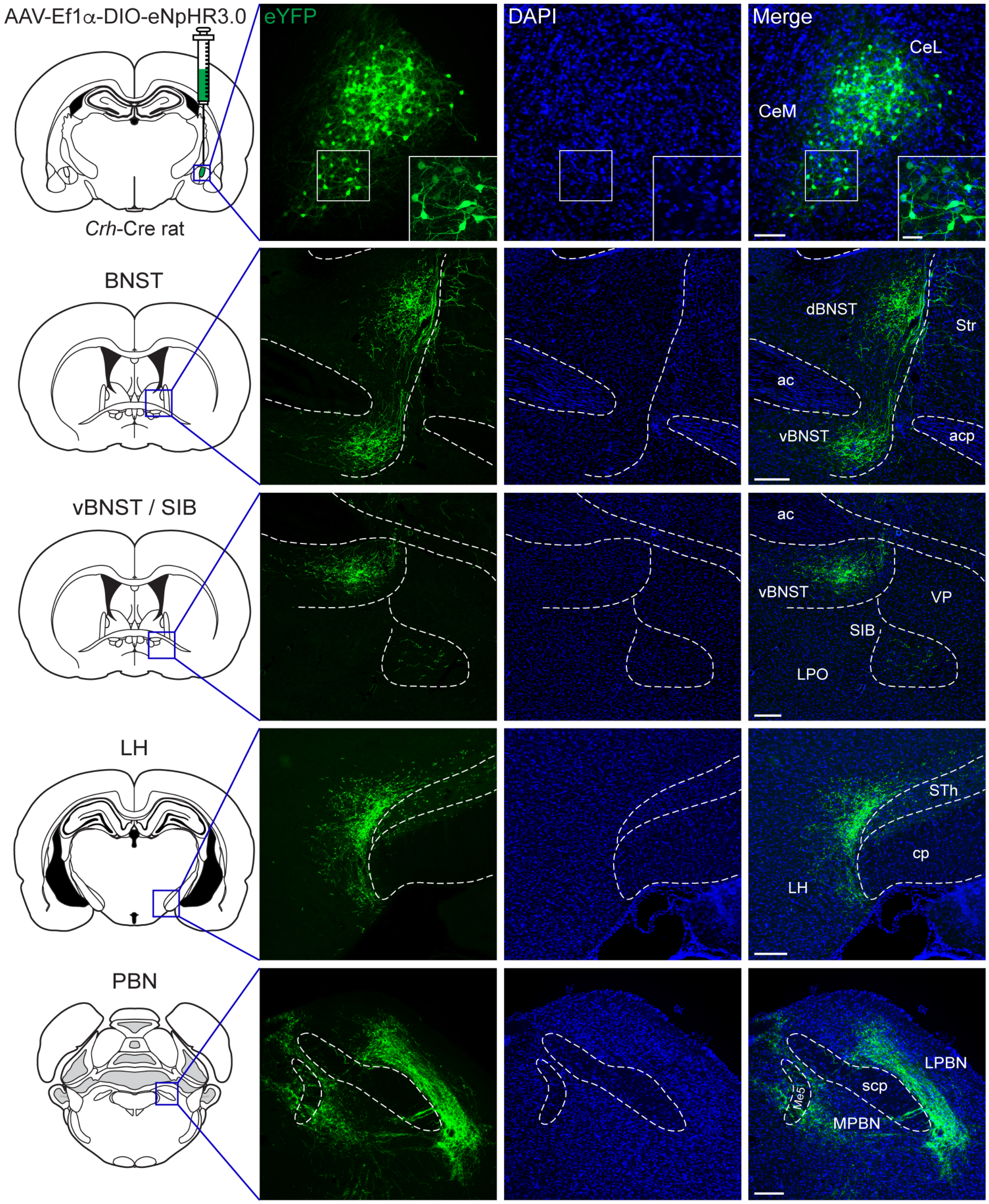

Supplementary Figure 3. Schematic diagram and representative images of the area of virus injection (CeA) and projection regions where fibers were placed for optogenetic inhibition (CeA, BNST, SI, LH, and PBN). Scalebars: 200 um; insert: 50 um.

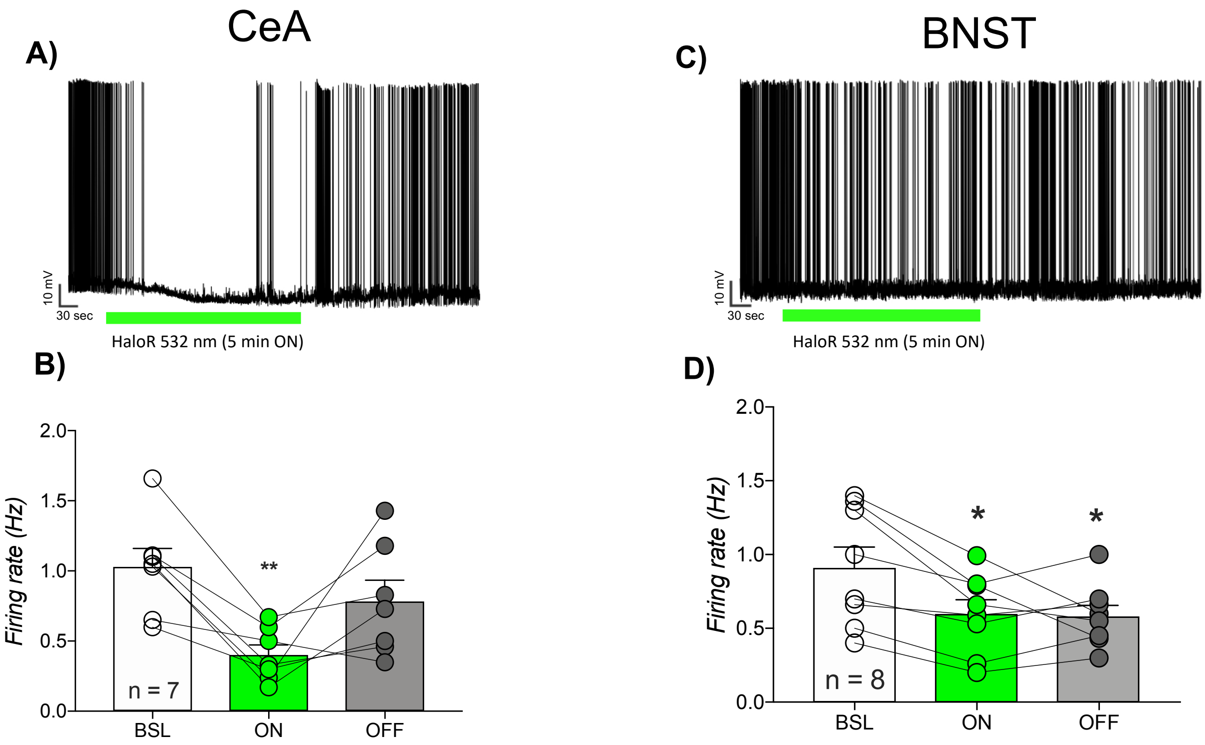

Supplementary Figure 4. A sustained 5 minute illumination in the CeA and BNST reduces cell firing in both regions. (**A**) Current-clamp recording of a CeA neuron: action potential firing was robustly decreased in response to the delivery of 5 min continuous green light (HaloR, 532 nm). (**B**) Continuous 5 min green light exposure of CRF neurons significantly reduced firing frequency ($F_{2,12} = 9.03$, $p < 0.001$) in the CeA. **$p < 0.01$ *vs* BSL and OFF. (**C**) Current-clamp recording of a BNST neuron: a 5min exposure to the green light (HaloR, 532 nm) decreased action potential firing. (**D**) Inhibition of $CRF^{CeA\text{-}BNST}$ terminals significantly reduced firing frequency ($F_{2,14} = 6.008$, $p < 0.05$) in the BNST. * $p < 0.05$ vs BSL. Data are expressed as ± SEM.

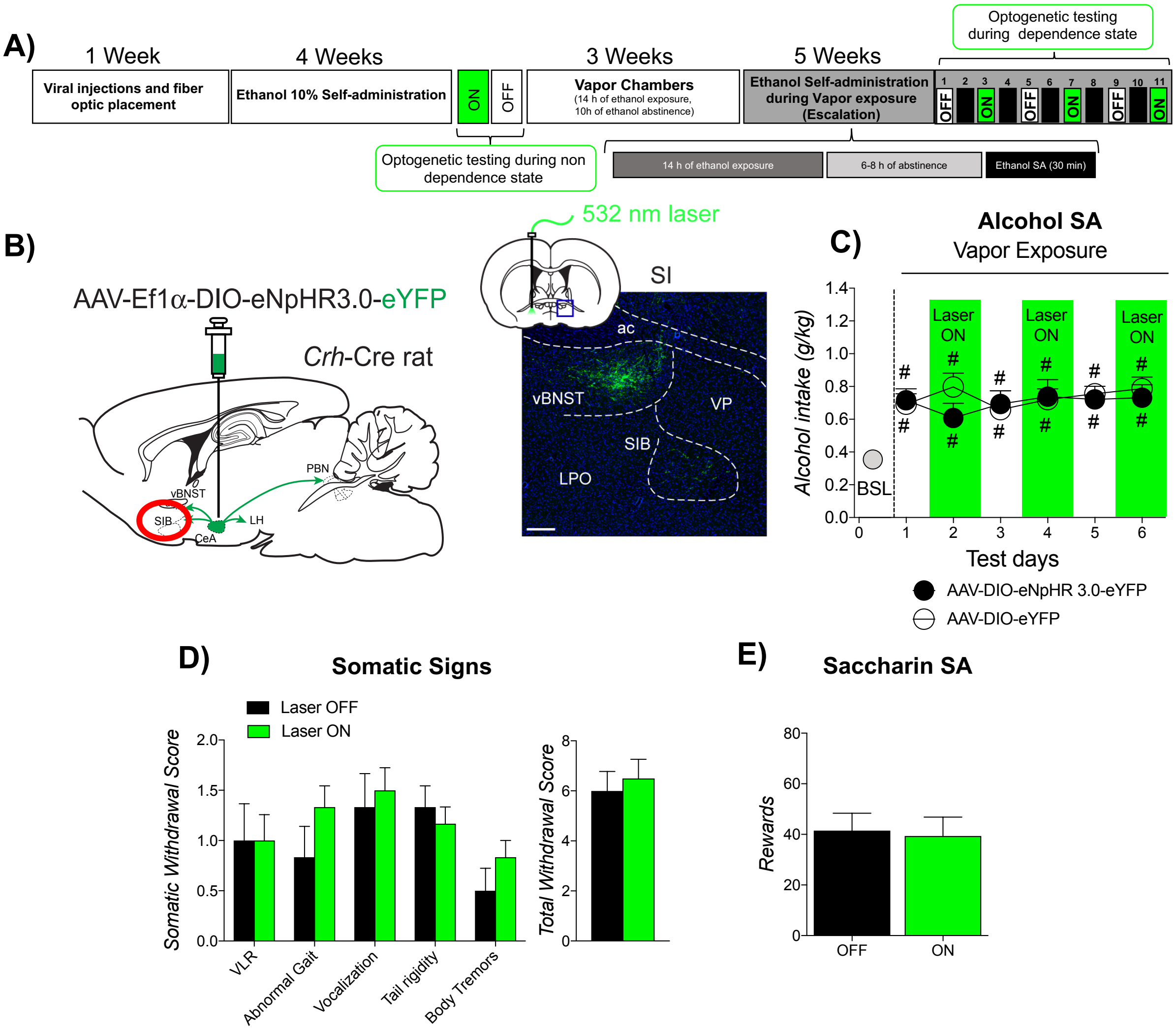

Supplementary Figure 5. (A) Timeline of the experiment. **(B)** Representative images of the area of injection (CeA) and area of optogenetic inhibition (SI). Scale bar: 200 um. (**C**) Black circles: effect of optogenetic inactivation of $CRF^{CeA\text{-}SI}$ terminals. The ANOVA revealed a significant effect of time ($F_{2,10} = 15.10$, $p < 0.001$) but no effect of laser ($F_{1,5} = 4.39$, $p > 0.05$) and no time × laser interaction ($F_{2,10} = 3.66$, $p > 0.05$). A separate one-way ANOVA indicated that the animals escalated their alcohol intake compared with baseline pre-vapor exposure ($F_{6,30} = 4.28$, $p < 0.01$) that was unaffected by the optogenetic inhibition of $CRF^{CeA\text{-}SI}$ terminals. Operant responding for alcohol remained high on both the laser ON and OFF days ($p < 0.05$ *vs*.

baseline;). $^{\#}p < 0.05$, *vs*. BSL. White circles: effect of green laser activation in the SI in control rats injected with AAV-DIO-eYFP in the CeA. The animals escalated their alcohol intake compared with baseline pre-vapor exposure ($F_{2,14} = 15.1$, $p < 0.001$) that was unaffected by the optogenetic inhibition of $CRF^{CeA\text{-}SI}$ terminals. Operant responding for alcohol remained high on both the laser ON and OFF days (both $p < 0.05$, *vs*. baseline). $^{\#}p < 0.05$, *vs*. BSL. (**D**) Effect of $CRF^{CeA\text{-}SI}$ terminal inhibition on somatic withdrawal signs. The inhibition of $CRF^{CeA\text{-}SI}$ terminals did not affect overall withdrawal severity ($U = 15.50$, $p > 0.05$). (**E**) Optogenetic inhibition of $CRF^{CeA\text{-}SI}$ terminals did not affect saccharin self-administration ($t_5 = 0.22$, $p > 0.05$). Data are expressed as ± SEM.

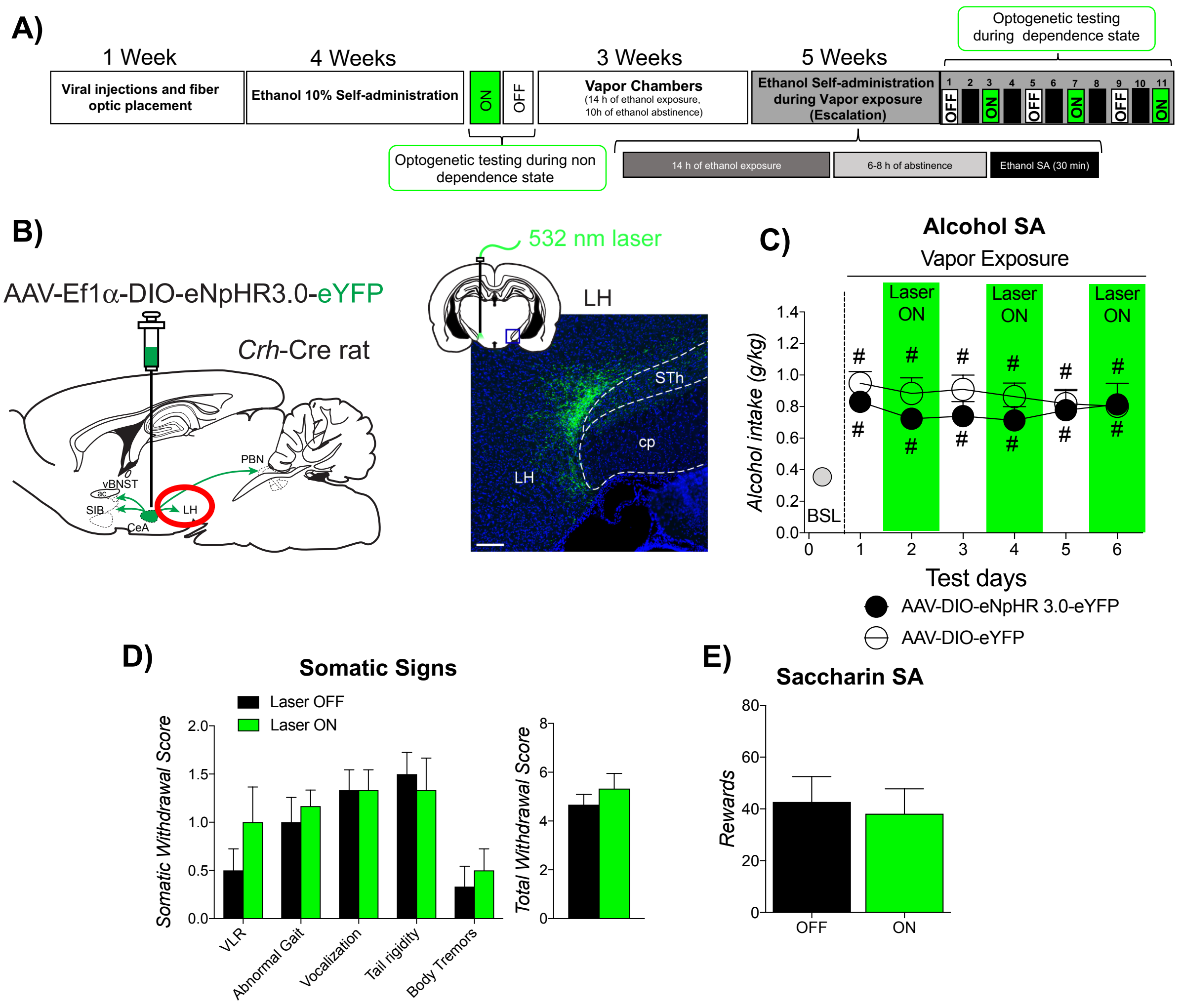

Supplementary Figure 6. (A) Timeline of the experiment. **(B)** Representative images of the area of injection (CeA) and area of optogenetic inhibition (LH/pSTN). Scale bar: 200 um. **(C)** Black circles: effect of optogenetic inactivation of $CRF^{CeA\text{-}LH/\ pSTN}$ terminals. The ANOVA revealed a significant effect of time ($F_{2,10} = 8.87$, $p < 0.01$) but no effect of laser ($F_{1,5} = 0.78$, $p > 0.05$) and no time × laser interaction ($F_{2,10} = 0.16$, $p > 0.05$). A separate one-way ANOVA demonstrated the escalation of alcohol intake compared with baseline pre-vapor exposure ($F_{6,30} = 4.28$, $p < 0.01$) that was unaffected by the optogenetic inhibition of $CRF^{CeA\text{-}LH/pSTN}$ terminals. Operant responding for alcohol remained high on both the laser ON and OFF days (both $p < 0.05$, *vs*. baseline). $^{\#}p < 0.05$, *vs*. BSL. White circles: effect of green laser activation in the LH-

pSTN in control rats that were injected with AAV-DIO-eYFP in the CeA. The animals escalated their alcohol intake compared with baseline pre-vapor exposure ($F_{2,14} = 8.58$, $p < 0.01$) that was unaffected by the optogenetic inhibition of $CRF^{CeA\text{-}LH/pSTN}$ terminals. Operant responding for alcohol remained high on both the laser ON and OFF days (both $p < 0.01$, *vs*. baseline). $^{\#}p < 0.05$, *vs*. BSL. (**D**) Effect of $CRF^{CeA\text{-}LH/\ pSTN}$ terminal inhibition on somatic withdrawal signs. The inhibition of $CRF^{CeA\text{-}LH/pSTN}$ terminals did not affect overall withdrawal severity ($U = 14.00$, $p > 0.05$). (**E**) Optogenetic inhibition of $CRF^{CeA\text{-}LH/pSTN}$ terminals did not affect saccharin self-administration ($t_5 = 1.024$, $p > 0.05$). Data are expressed as ± SEM.

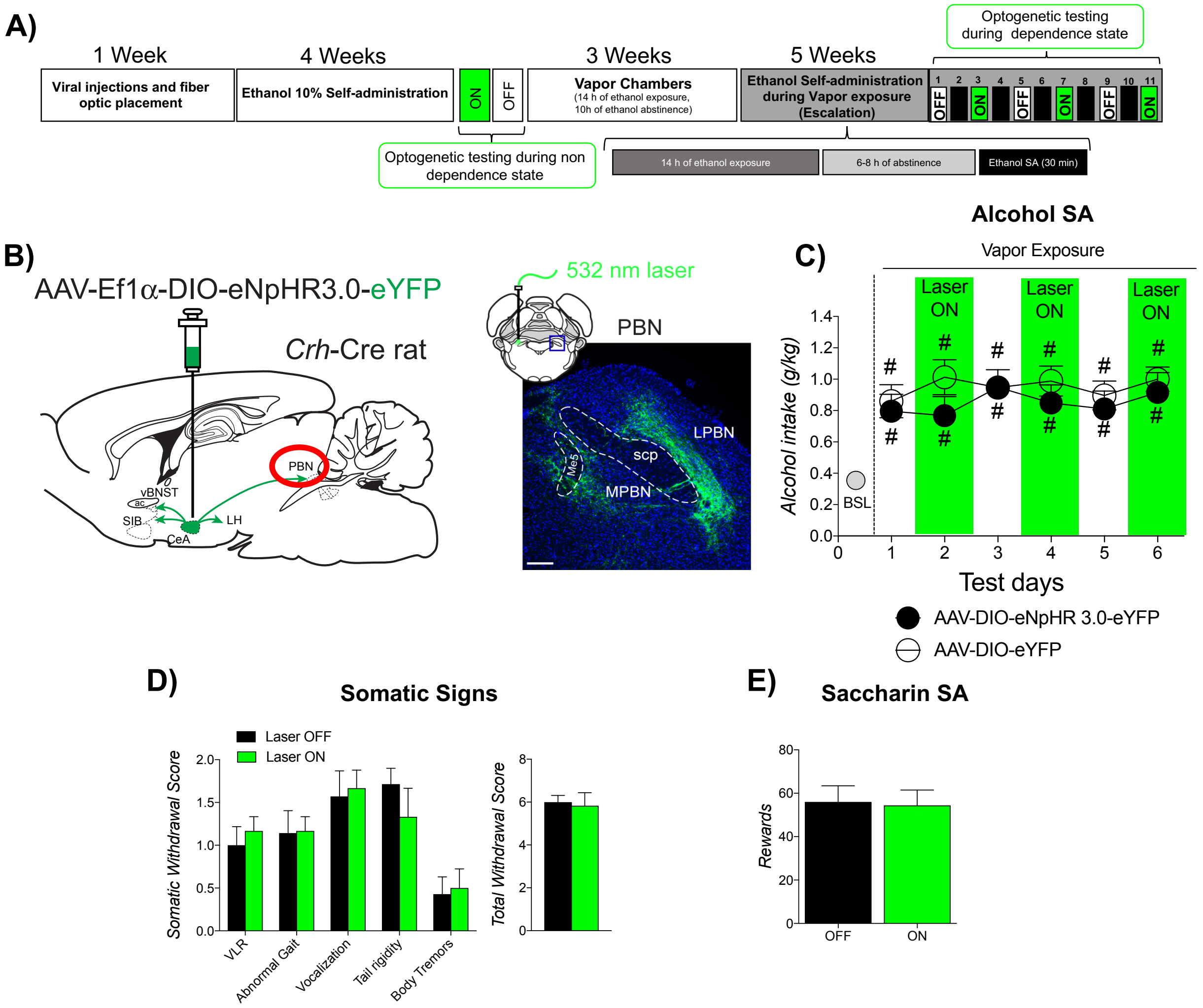

Supplementary Figure 7. (A) Timeline of the experiment. **(B)** Representative images of the area of injection (CeA) and area of optogenetic inhibition (PBN). Scale bar: 200 um. (**C**) Black circles: effect of optogenetic inactivation of $CRF^{CeA\text{-}PBN}$ terminals. The ANOVA revealed a significant effect of time ($F_{2,22} = 7.14$, $p < 0.01$) but no effect of laser ($F_{1,11} = 0.73$, $p > 0.05$) and no time × laser interaction ($F_{2,22} = 1.99$, $p > 0.05$). A separate one-way ANOVA indicated the escalation of alcohol intake compared with baseline pre-vapor exposure ($F_{6,66} = 4.75$, $p < 0.001$) that was unaffected by the optogenetic inhibition of $CRF^{CeA\text{-}PBN}$ terminals. $^{\#}p < 0.05$ *vs*. BSL. White circles: effect of green laser activation in the PBN in control rats injected with AAV-DIO-eYFP in the CeA. The animals escalated their alcohol intake compared with baseline pre-vapor

exposure ($F_{2,14} = 5.21$, $p < 0.05$) that was unaffected by the optogenetic inhibition of $CRF^{CeA\text{-}PBN}$ terminals. Operant responding for alcohol remained high on both the laser ON and OFF days (both $p < 0.05$, *vs*. baseline). $^{\#}p < 0.05$, *vs*. BSL. (**D**) Effect of $CRF^{CeA\text{-}PBN}$ terminal inhibition on somatic withdrawal signs. The inhibition of $CRF^{CeA\text{-}PBN}$ terminals did not affect overall withdrawal severity ($U = 18.50$, $p > 0.05$). (**E**) Optogenetic inhibition of $CRF^{CeA\text{-}PBN}$ terminals did not affect saccharin self-administration ($t_{11} = 0.77$, $p > 0.05$). Data are expressed as ± SEM.

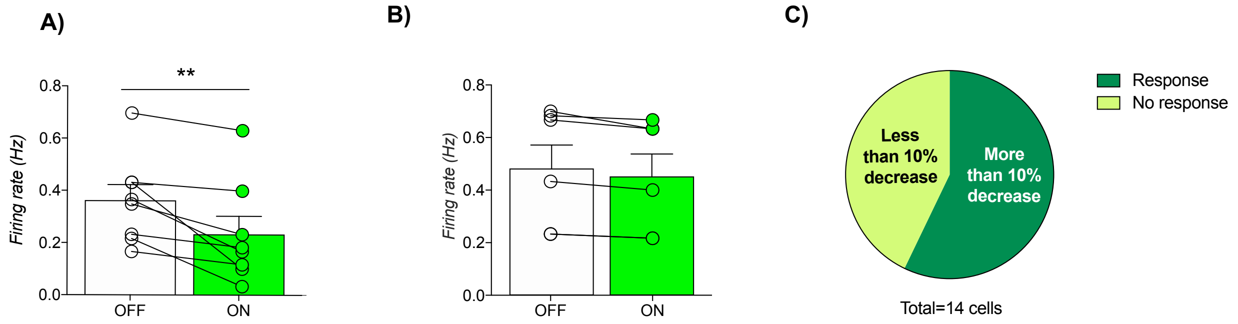

Supplementary Figure 8. (**A**) Inhibition of CRF$^{CeA\text{-}BNST}$ terminals significantly reduced firing frequency ($t_8 = 5.042$, $p < 0.01$) in 8 BNST cells out of a total of 14 cells recorded. $^{**}p < 0.01$ *vs*. OFF. (**B**) Inhibition of CRF$^{CeA\text{-}BNST}$ terminals did not alter the firing frequency in 6 BNST cells out of a total of 14 cells recorded. (**C**) Neurons that showed less than a 10% decrease to the CRF$^{CeA\text{-}BNST}$ inhibition were considered "non-responders" (n=6) while the other population was considered "responders" (n=8).

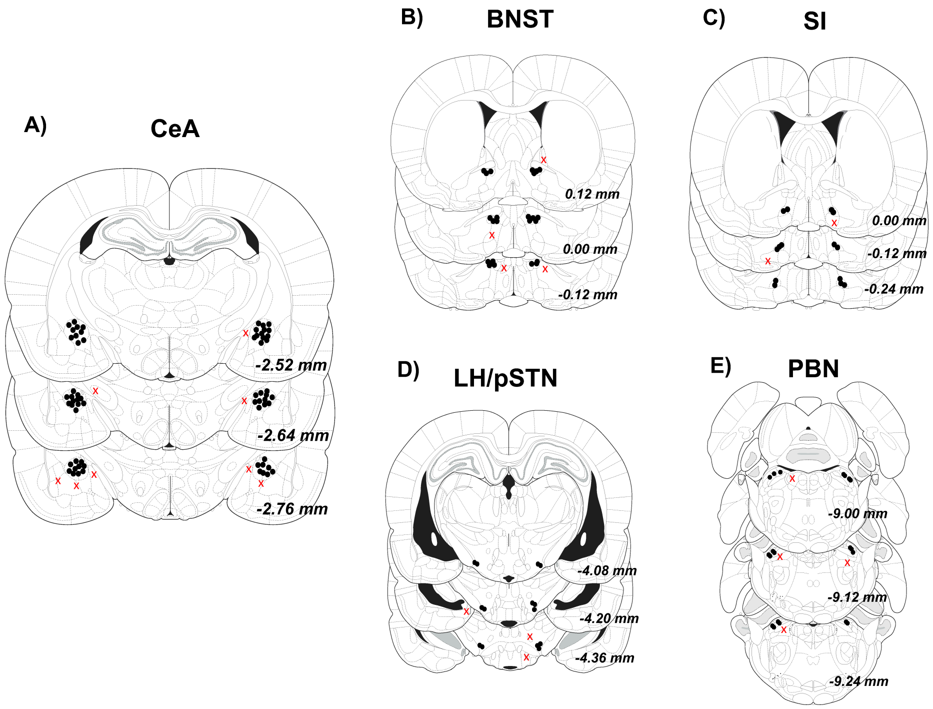

Supplementary Figure 9. Schematic illustrations of the virus injections and optical fiber placements. Black dots represent correct fiber placements. Rats with incorrect fiber placements (red X) were excluded from the analysis.
