# Supplementary material for: Inactivation of a CRF-dependent amygdalofugal pathway reverses addiction-like behaviors in alcohol-dependent rats
Source: Nat Commun. 2019 Mar 18;10:1238. doi: 10.1038/s41467-019-09183-0 (PMC6423296; doi:10.1038/s41467-019-09183-0)
Supplement: Supplementary file 3 — Description of Additional Supplementary Files [file 41467_2019_9183_MOESM3_ESM.pdf]

Description of Additional Supplementary Files

File Name: Supplementary Movie 1
Description: 3d reconstruction at 63X magnification of CeA CRF neurons
