# Supplementary material for: Inactivation of a CRF-dependent amygdalofugal pathway reverses addiction-like behaviors in alcohol-dependent rats
Source: Nat Commun. 2019 Mar 18;10:1238. doi: 10.1038/s41467-019-09183-0 (PMC6423296; doi:10.1038/s41467-019-09183-0)
Supplement: Supplementary file 5 — Reporting Summary [file 41467_2019_9183_MOESM5_ESM.pdf]

nature research

Corresponding author(s): Olivier George

Last updated by author(s): 2019/02/13

Reporting Summary

Nature Research wishes to improve the reproducibility of the work that we publish. This form provides structure for consistency and transparency in reporting. For further information on Nature Research policies, see Authors & Referees and the Editorial Policy Checklist.

Statistics

For all statistical analyses, confirm that the following items are present in the figure legend, table legend, main text, or Methods section.

n/a	Confirmed	
☐	☒	The exact sample size (n) for each experimental group/condition, given as a discrete number and unit of measurement
☐	☒	A statement on whether measurements were taken from distinct samples or whether the same sample was measured repeatedly
☐	☒	The statistical test(s) used AND whether they are one- or two-sided<br>*Only common tests should be described solely by name; describe more complex techniques in the Methods section.*
☒	☐	A description of all covariates tested
☒	☐	A description of any assumptions or corrections, such as tests of normality and adjustment for multiple comparisons
☐	☒	A full description of the statistical parameters including central tendency (e.g. means) or other basic estimates (e.g. regression coefficient) AND variation (e.g. standard deviation) or associated estimates of uncertainty (e.g. confidence intervals)
☐	☒	For null hypothesis testing, the test statistic (e.g. F, t, r) with confidence intervals, effect sizes, degrees of freedom and P value noted<br>*Give P values as exact values whenever suitable.*
☒	☐	For Bayesian analysis, information on the choice of priors and Markov chain Monte Carlo settings
☒	☐	For hierarchical and complex designs, identification of the appropriate level for tests and full reporting of outcomes
☒	☐	Estimates of effect sizes (e.g. Cohen's d, Pearson's r), indicating how they were calculated

Our web collection on statistics for biologists contains articles on many of the points above.

Software and code

Policy information about availability of computer code

Data collection	Data were collected using the Med PC software from Med associated (for animal studies). For the immunohistochemistry Stitched z-series images of the entire CeA were imported into Imaris software (Bitplane-Andor) and Fiji for quantification.
Data analysis	Data were analyzed using Statistica 7 software.

For manuscripts utilizing custom algorithms or software that are central to the research but not yet described in published literature, software must be made available to editors/reviewers. We strongly encourage code deposition in a community repository (e.g. GitHub). See the Nature Research guidelines for submitting code & software for further information.

Data

Policy information about availability of data

All manuscripts must include a data availability statement. This statement should provide the following information, where applicable:

- Accession codes, unique identifiers, or web links for publicly available datasets
- A list of figures that have associated raw data
- A description of any restrictions on data availability

The datasets generated during and/or analyzed during the current study are available from the corresponding author on reasonable request.

Field-specific reporting

Please select the one below that is the best fit for your research. If you are not sure, read the appropriate sections before making your selection.

☒ Life sciences ☐ Behavioural & social sciences ☐ Ecological, evolutionary & environmental sciences

Life sciences study design

All studies must disclose on these points even when the disclosure is negative.

Sample size	We selected the number of animals used in each experiment (>8/group) based on a power analysis; this sample size will provide at least 80% power to detect effect sizes (Cohen's d), ranging from d = 1.2 to 2.0 at a significance level of $p < 0.05$.
Data exclusions	No data were excluded from the analysis
Replication	All attempts at replication were successful.
Randomization	Groups were leveled based on the animals' alcohol self-administration baseline.
Blinding	The investigators were blind to the experimental conditions

Reporting for specific materials, systems and methods

We require information from authors about some types of materials, experimental systems and methods used in many studies. Here, indicate whether each material, system or method listed is relevant to your study. If you are not sure if a list item applies to your research, read the appropriate section before selecting a response.

Materials & experimental systems

n/a	Involved in the study	
☐	☒	Antibodies
☒	☐	Eukaryotic cell lines
☒	☐	Palaeontology
☐	☒	Animals and other organisms
☒	☐	Human research participants
☒	☐	Clinical data

Methods

n/a	Involved in the study	
☒	☐	ChIP-seq
☒	☐	Flow cytometry
☒	☐	MRI-based neuroimaging

Antibodies

Antibodies used

rabbit monoclonal anti-Fos antibody (Cell Signaling Technology, catalog no. 2250), anti-Fos (1:500, Millipore, catalog no. AB4532) and anti-NeuN (1:1000, Millipore, catalog no. MAB377), Anti-CRF (1:1000, Santa Cruz biotechnology, catalog no. SC-1761)

Validation

Anti-cFos (1:500, Millipore, catalog no. AB4532 (ABE457))

http://www.emdmillipore.com/US/en/product/Anti-c-Fos-Antibody,MM_NF-ABE457

Validation:

Immunohistochemistry Analysis: A 1:1,000 dilution from a representative lot detected c-Fos in rat pons and rat cerebellum tissues.

Evaluated by Western Blot in PMA(TPA) treated HeLa cell lysate.

Western Blot Analysis: 0.5 µg/mL of this antibody detected c-Fos in 10 µg of PMA(TPA) treated HeLa cell lysate.

References:

Suckling-induced Fos activation and melanin-concentrating hormone immunoreactivity during late lactation. Alvisi, RD; Diniz, GB; Da-Silva, JM; Bittencourt, JC; Felicio, LF Life sciences 241-6 2016

Antinociceptive and hypnotic activities of pregabalin in a neuropathic pain-like model in mice.
Wang, TX; Yin, D; Guo, W; Liu, YY; Li, YD; Qu, WM; Han, WJ; Hong, ZY; Huang, ZL
Pharmacology, biochemistry, and behavior 31-9 2015

Gelsemine alleviates both neuropathic pain and sleep disturbance in partial sciatic nerve ligation mice. Wu, YE; Li, YD; Luo, YJ; Wang, TX; Wang, HJ; Chen, SN; Qu, WM; Huang, ZL
Acta pharmacologica Sinica 1308-17 2015

Leptin signaling in astrocytes regulates hypothalamic neuronal circuits and feeding.
Kim, JG; Suyama, S; Koch, M; Jin, S; Argente-Arizon, P; Argente, J; Liu, ZW; Zimmer, MR; Jeong, JK; Szigeti-Buck, K; Gao, Y; Garcia-

Caceres, C; Yi, CX; Salmaso, N; Vaccarino, FM; Chowen, J; Diano, S; Dietrich, MO; Tschöp, MH; Horvath, TL
Nature neuroscience 17 908-10 2014

Anti-NeuN (1:1000, Millipore, catalog no. MAB377)

http://www.emdmillipore.com/US/en/product/Anti-NeuN-Antibody-clone-A60,MM_NF-MAB377

https://www.biocompare.com/9776-Antibodies/62926-AntiNeuN-clone-A60/

Clone A60

Routinely evaluated by immunohistochemistry on brain tissue.

Immunohistochemistry(paraffin) Analysis:
NeuN (cat. # MAB377) staining pattern/morphology in rat cerebellum. Tissue pretreated with Citrate, pH 6.0. This lot of antibody was diluted to 1:100, using IHC-Select® Detection with HRP-DAB. Immunoreactivity is seen as nuclear staining in the neurons in the granular layer. Note that there is no signal detected in the nucleus of Purkinje cells.
Optimal Staining With Citrate Buffer, pH 6.0, Epitope Retrieval: Rat Cerebellum

Immunohistochemistry:
1:100-1:1,000. The antibody works best on polyester wax embedded tissue but also works on paraffin embedded tissue at a lower working dilution. The antibody works well with formaldehyde-based fixatives. Citric acid and microwave pretreatment has been used successfully (Sarnat, 1998).
References
Bolos M, Spuch C, Ordoñez-Gutierrez L, Wandosell F, Ferrer I, Carro E. Neurogenic effects of β-amyloid in the choroid plexus epithelial cells in Alzheimer's disease. Cell Mol Life Sci. 2013 Aug;70(15):2787-97.

Jäger C, Lendvai D, Seeger G, Brückner G, Matthews RT, Arendt T, Alpár A, Morawski M. Perineuronal and perisynaptic extracellular matrix in the human spinal cord. Neuroscience. 2013 May 15;238:168-84.

Seelke AM, Dooley JC, Krubitzer LA. Differential changes in the cellular composition of the developing marsupial brain. J Comp Neurol. 2013 Aug 1;521(11):2602-20.

Trabalza A, Georgiadis C, Eleftheriadou I, Hislop JN, Ellison SM, Karavassilis ME, Mazarakis ND. Venezuelan equine encephalitis virus glycoprotein pseudotyping confers neurotropism to lentiviral vectors. Gene Ther. 2013 Jul;20(7):723-32

Anti-CRF (1:1000, Santa Cruz biotechnology, catalog no. SC-1761)

https://www.citeab.com/antibodies/784033-sc-1761-crf-s-19

Validated in transgenic animals, WB, IF, and IHC.
References
Pomrenze MB, Millan EZ, Hopf FW,Keiflin R, Maiya R, Blasio A, Dadgar J,Kharazia V, De Guglielmo G,Crawford E, Janak PH, George O,Rice KC and Messing RO (2015) ATransgenic Rat for Investigating the Anatomy and Function of Corticotrophin Releasing Factor Circuits. Front. Neurosci. 9:487.doi: 10.3389/fnins.2015.00487

Kurada L, Yang C, Lei S. Corticotropin-releasing factor facilitates epileptiform activity in the entorhinal cortex: roles of CRF2 receptors and PKA pathway. PLoS ONE. 2014;9:e88109

Grieder T, Herman M, Contet C, Tan L, Vargas-Perez H, Cohen A, et al. VTA CRF neurons mediate the aversive effects of nicotine withdrawal and promote intake escalation. Nat Neurosci. 2014;17:1751-8

Zmijewski M, Sharma R, Slominski A. Expression of molecular equivalent of hypothalamic-pituitary-adrenal axis in adult retinal pigment epithelium. J Endocrinol. 2007;193:157-69

Animals and other organisms

Policy information about studies involving animals; ARRIVE guidelines recommended for reporting animal research

Laboratory animals	Adult male Crh-Cre rats, 2 months old and weighing 200-225 g at the beginning of the experiments, were housed in groups of two per cage (self-administration groups) in a temperature-controlled (22°C) vivarium on a 12 h/12 h light/dark cycle (lights on at 10:00 PM) with ad libitum access to food and water. All of the behavioral tests were conducted during the dark phase of the light/dark cycle.
Wild animals	N/A
Field-collected samples	N/A
Ethics oversight	All of the procedures adhered to the National Institutes of Health Guide for the Care and Use of Laboratory Animals and were approved by the Institutional Animal Care and Use Committee of The Scripps Research Institute.

Note that full information on the approval of the study protocol must also be provided in the manuscript.
